# Latissimus Dorsi Musculocutaneous Flap for Complex Breast Reconstruction: Indications, Outcomes and a Proposed Algorithm

**DOI:** 10.1097/GOX.0000000000002382

**Published:** 2019-08-08

**Authors:** George Kokosis, Nima Khavanin, Maurice Y. Nahabedian

**Affiliations:** From the * Department of Plastic and Reconstructive Surgery, Johns Hopkins University, Baltimore, Md.; † National Center for Plastic Surgery, McLean, Va.

## Abstract

The latissimus dorsi (LD) flap is an ideal flap for salvage following failed primary breast reconstruction in the setting of radiation therapy. This study will review outcomes following secondary reconstruction with the LD flap with or without a tissue expander (TE) or implant (I).

**Methods:**

The following 4 cohorts were included: 1-stage LD only in 28 patients (48.3%), 1-stage LD + I in 7 patients (12.1%), 2-stage LD + TE/I in 8 patients (13.8%), and 3-stage LD + TE + I in 15 patients (25.9%).

**Results:**

The average age across all patients was 53.2 years. Complications did not differ significantly across the 4 cohorts. Complications included partial flap necrosis, wound dehiscence, seroma, and infection occurring in 4 of 28 patients of 1-stage LD alone, 2 of 7 (28.6%) patients of 1-stage LD + I, 5 of 8 (52.5%) patients of 2-stage LD + TE/I, and 4 of 15 (26.7%) patients of 3-stage LD + TE + I (*P* = 0.055). Reoperation rates were 10.7%, 14.3%, 25%, and 0% across the 4 cohorts, respectively (*P* = 0.295). The LD only cohort had a 14.3% surgical revision rate, compared with 42.9% in the 1-stage + I, 50% in the 2-stage + TE/I, and 33.3% in the 3-stage LD + TE + I (*P* = 0.135). The rate of contralateral symmetry procedures was 10.7%, 0%, 25%, and 6.7%, across the 4 cohorts, respectively (*P* = 0.410).

**Conclusion:**

Secondary breast reconstruction with the LD flap in 1, 2, or 3 stages has demonstrated success. A decision-making algorithm is provided.

## INTRODUCTION

The latissimus dorsi (LD) musculocutaneous flap was first introduced in the 1970s and has remained a viable option for women seeking breast reconstruction following partial and total mastectomy.^1,2^ The reasons for its longevity are due to a variety of reasons; however, its consistent and reliable vascular pedicle and the ease of harvest have contributed to its success and made it popular among reconstructive surgeons.^3–5^ Its utility in breast reconstruction has been well documented for both immediate and delayed reconstructions.^3,6^ With the advent of perforator-based flaps, many plastic surgeons have abandoned immediate breast reconstruction with the LD musculocutaneous flap in lieu of preserving the donor site muscle to avoid the adverse effects related to muscle sacrifice.^7,8^ However, there are circumstances in which the use of this flap is beneficial, especially in situations of prior reconstructive failure,^9^ previous radiation therapy (RT),^10^ recurrent cancer after breast conservation therapy,^11^ and implant infection.^12^

Secondary or delayed breast reconstruction with the LD flap is beneficial for several reasons. It is associated with few complications, it does not require microvascular anastomosis, and it can provide well-vascularized tissue to a previously radiated chest wall. The LD flap can be used with or without prosthetic devices that can be placed simultaneously or on a staged basis.^13,14^

The purpose of this article is to review the primary author’s experience using the LD musculocutaneous flap as a means of salvaging breast reconstruction in complex patients who have had prior reconstructive failure, previous radiation, and/or infection. Our hypothesis is that future reconstructive failure and complications can be minimized using the staged approach and that placing well-vascularized tissue over a previously infected or radiated chest wall will improve the quality of the adjacent or overlying tissues and facilitate reconstructive success. Four reconstructive cohorts will be analyzed and compared that include: (1) LD flap alone, (2) LD flap and immediate implant, (3) LD flap and delayed implant (2-stage reconstruction), and (4) LD flap and delayed tissue expander (TE) and delayed implant reconstruction (3-stage reconstruction).

## METHODS

This was a retrospective review of a prospectively maintained database of the senior authors’ patients who underwent immediate or delayed breast reconstruction with the LD musculocutaneous flap in the setting of previous chest wall RT associated with prior reconstructive failure with a microvascular free tissue transfer, nonhealing chest wall wounds, and prosthetic failure following infection. The LD flap reconstruction was performed with or without TEs and implants in 1, 2, or 3 stages. Patient’s history, operative details, and surgical outcomes were collected for all patients over an 18-year period (1998–2016).

Preoperative variables included patient’s age, diabetes mellitus, timing of reconstruction relative to mastectomy, radiation history, previous attempts at reconstruction, and the presence of a chronic chest wound. Outcomes included postoperative complications, such as Baker grade 3 or 4 capsular contracture, mastectomy flap necrosis, wound dehiscence, implant exposure, seroma, hematoma, partial flap loss, infection, implant malposition, explantation, and need for reoperation, and aesthetic outcomes, including contralateral symmetry procedures and aesthetic revisions. Overall complication was defined as the presence of any of the above postoperative complications.

Descriptive statistics was calculated for each of the 4 cohorts and compared among one another using ANOVA for continuous variables and *χ*^2^ tests for nominal variables. Complication rates and aesthetic procedures were compared across the cohorts using *χ*^2^ tests. All analyses were performed using SPSS v21.0 (IBM Corp, Armonk, NY).

### Algorithm

**Fig. 1.**
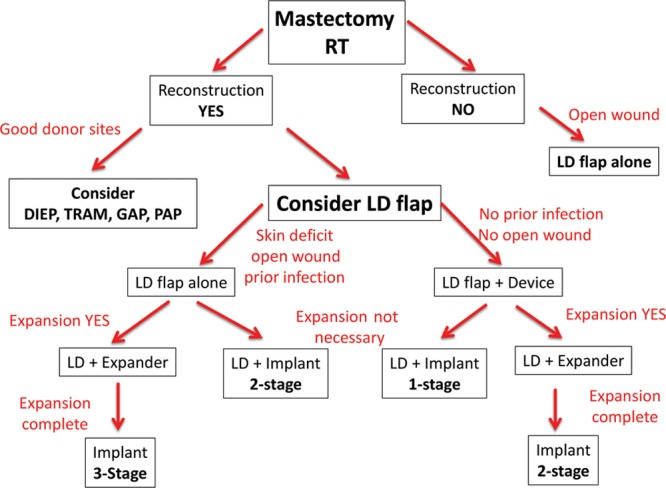
Treatment algorithm for salvage latissimus dorsi breast reconstruction. DIEP, deep inferior epigastric perforator flap; TRAM, transverse rectus abdominis musculocutaneous flap; GAP, gluteal artery perforator flap; and PAP, profunda artery perforator flap.

The decision to use the LD musculocutaneous flap was based on limited options following prior reconstructive failure in the setting of RT. Details of the algorithm are highlighted in Figure 1.

In patients who had prior mastectomy and RT, delayed prosthetic reconstruction was not offered. When the abdominal donor site was intact, an abdominal free flap was usually recommended and performed; however, in the event that an abdominal flap was not possible, then, alternative donor sites such as the LD musculocutaneous flap were considered. Other microvascular donor sites such as the gluteal and thigh regions are usually discussed; however, in this series of patients, all chose to have the LD flap. In patients who had prior microvascular free flap failure where the recipient vessels were not available, the LD flap was our preferred option. In patients who had a chronic chest wall wound and were not interested in formal breast reconstruction, then an LD musculocutaneous flap without a prosthetic device was recommended. In patients with a chronic wound, prior implant infection with explantation, or severe radiation changes, the LD musculocutaneous flap reconstruction was usually recommended and performed in 1, 2, or 3 stages depending on the degree of the deformity, surgeon judgment, and quality/quantity of the surrounding and available soft tissues. In some patients, the LD musculocutaneous flap and implant were performed in a single stage, and in other patients, it is performed in 2 stages. In the most complex patients, the reconstruction was performed in 3 stages, whereby the LD musculocutaneous flap was performed first followed by insertion of a TE and finally by removal of the TE and insertion of a permanent implant.

Incision choice for the LD flap was based on the pinch test to determine the optimal orientation of the skin territory to maximize soft-tissue volume. The LD muscle is not usually denervated. Immediate fat grafting of the LD muscle was not performed; however, delayed fat grafting was considered as a revisionary procedure. The timing between these sequential operations was usually 3–6 months. Optimal timing for placement of a permanent prosthetic device was when the LD flap was well healed, soft, and supple. It did not occur before healing was established. This required up to 12 months in some cases. Prosthetic devices were placed above the radiated pectoralis major muscle and under the nonradiated LD muscle.

## RESULTS

Overall, 58 patients met inclusion criteria, all of whom had undergone preoperative RT. Of them, 28 (48.3%) patients underwent 1-stage reconstruction with the LD musculocutaneous flap only, 7 (12.1%) patients underwent 1-stage LD musculocutaneous flap reconstruction with an implant, 8 (13.8%) patients underwent 2-stage LD musculocutaneous flap with a TE and implant, and 15 (25.9%) patients underwent 3-stage LD musculocutaneous flap with a delayed TE followed by permanent implant insertion. The mean age for all patients was 53.2 years (range 38–77 y). Comorbidities were uncommon and included diabetes mellitus (n = 1). No patient was actively using tobacco products (Table 1). Most patients underwent delayed reconstruction, and timing did not significantly differ across cohorts. The rate of failed previous reconstruction was significantly lower in the single-stage LD flap only cohort (10.7%) compared with the 1- (57.1%), 2- (62.5%), or 3-stage (46.7%) cohorts using an LD musculocutaneous flap with an implant (*P* = 0.003).

**Table 1.** Clinical Characteristics of the Patients Who Had Latissimus Dorsi Musculocutaneous Flap Reconstruction

	1-Stage Latissimus Only n = 28 48.30%	1-Stage Latissimus with Implant n = 7 12.10%	2-Stage Latissimus with TE/Implant n = 8 13.80%	3-Stage Latissimus with Delayed TE/Implant n = 15 25.90%	*P*
Age (y)*	55.5 ± 10.9	46.4 ± 9.8	55.3 ± 8.7	50.9 ± 8.6	0.133
Diabetic	0 (0%)	0 (0%)	0 (0%)	1 (6.7%)	–
Active smoker	0 (0%)	0 (0%)	0 (0%)	0 (0%)	–
Timing, relative to mastectomy					0.274
Immediate	2 (7.1%)	2 (28.6%)	0 (0%)	2 (13.3%)	
Delayed	26 (92.9%)	5 (71.4%)	8 (100%)	13 (86.7%)	
Previously attempted reconstruction	3 (10.7%)	4 (57.1%)	5 (62.5%)	7 (46.7%)	0.006
Type of prior reconstruction					0.003
Autologous	3 (100%)	1 (25%)	2 (40%)	0 (0%)	
Prosthetic	0 (0%)	3 (75%)	3 (60%)	7 (100%)	
Chronic chest wound	7 (25%)	0 (0%)	0 (0%)	0 (0%)	–

Complications were uncommon, and rates did not differ significantly across the 4 cohorts, both overall and with regard to any individual complication (Table 2). Four of 28 (14.3%) 1-stage LD musculocutaneous flap only patients experienced a complication, including partial flap necrosis, wound dehisce, and infection. Two of 7 (28.6%) 1-stage LD musculocutaneous flap with implant patients experienced a complication, compared with 5 of 8 (52.5%) patients with 2-stage procedures and 4 of 15 (26.7%) patients with 3-stage procedures (*P* = 0.055). Reoperation rates were 10.7%, 14.3%, 25%, and 0% across the 4 cohorts, respectively (*P* = 0.295).

**Table 2.** Complication Rates Following 1-, 2-, and 3-Stage Latissimus Dorsi Musculocutaneous Flap Reconstruction

	1-Stage Latissimus Only n = 28 48.30%	1-Stage Latissimus with Implant n = 7 12.10%	2-Stage Latissimus with TE/Implant n = 8 13.80%	3-Stage Latissimus with Delayed TE/Implant n = 15 25.90%	*P*
Any complication	4 (14.3%)	2 (28.6%)	5 (52.5%)	4 (26.7%)	0.055
Capsular contracture, grades 3 and 4	–	1 (14.3%)	2 (25%)	1 (6.7%)	0.467
Mastectomy flap necrosis	1 (3.6%)	0	0	0	0.779
Wound dehiscence	1 (3.6%)	0	0	0	0.779
Implant exposure	–	1 (14.3%)	0	0	0.183
Seroma, any	0	0	2 (25%)	2 (12.3%)	0.054
Seroma, breast	0	0	1 (12.5%)	1 (6.7%)	0.297
Seroma, back	0	0	1 (12.5%)	1 (6.7%)	0.297
Hematoma	1 (3.6%)	0	0	0	0.779
Partial flap loss	1 (3.6%)	0	0	0	0.779
Infection	1 (3.6%)	0	1 (12.5%)	1 (6.7%)	0.689
Implant malposition	–	0	1 (12.5%)	0	0.241
Explantation	–	1 (14.3%)	1 (12.5%)	0	0.339
Reoperation	3 (10.7%)	1 (14.3%)	2 (25%)	0	0.295

**Fig. 2.**
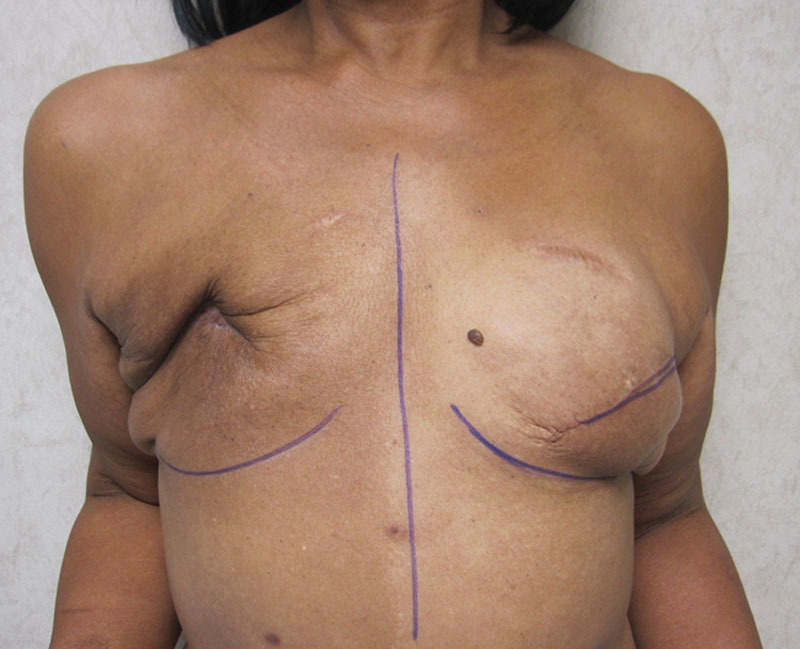
Preoperative photograph following radiation therapy and failed prosthetic reconstruction to the right breast.

**Fig. 3.**
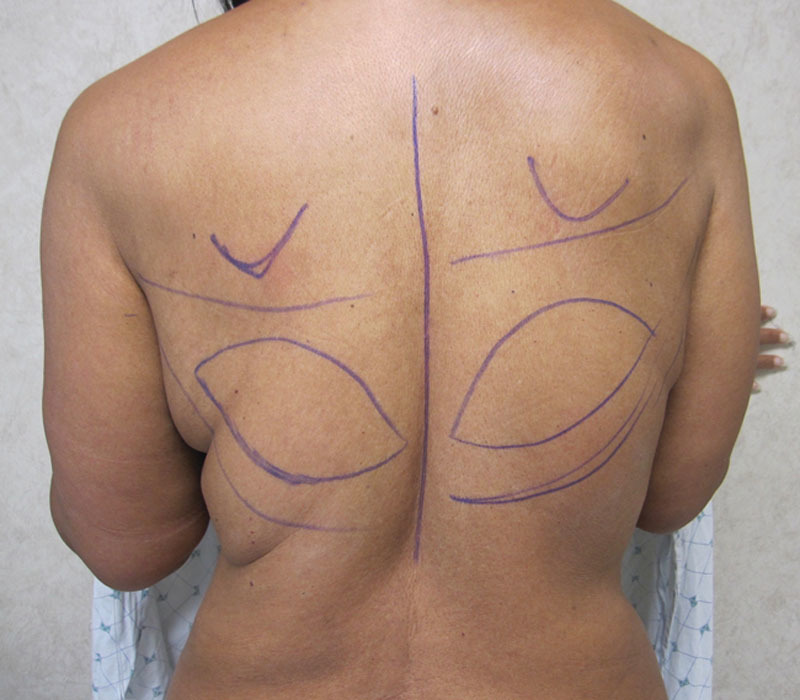
Preoperative markings in preparation for the stage 1 bilateral latissimus dorsi musculocutaneous flap breast reconstruction.

**Fig. 4.**
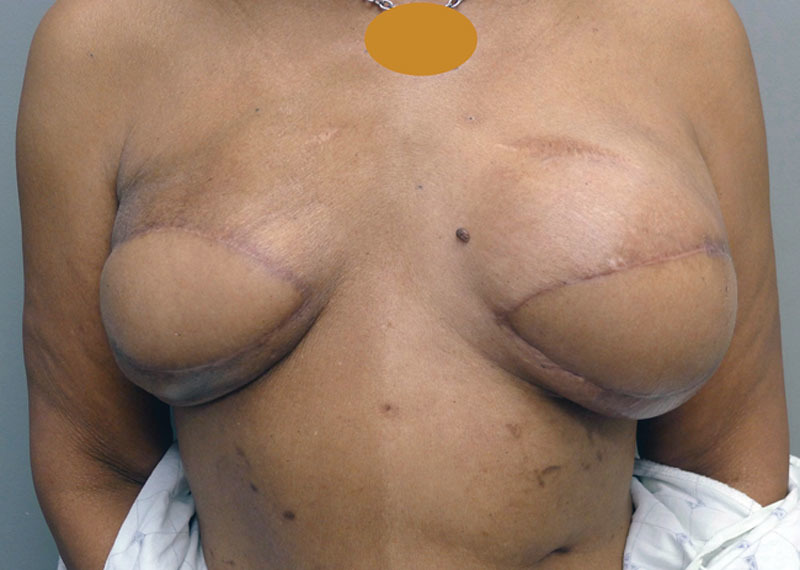
Postoperative photography following stage 3 insertion of bilateral 330 cm^3^ shaped permanent silicone gel implants demonstrating excellent volume and contour symmetry.

**Fig. 5.**
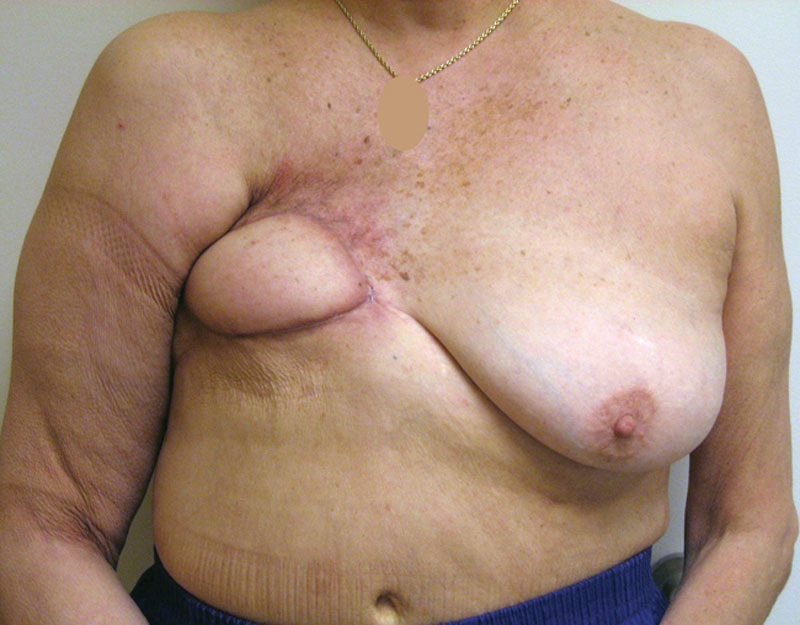
Preoperative image of a woman following mastectomy and radiation with a chronic, poorly healing chest wall wound.

**Fig. 6.**
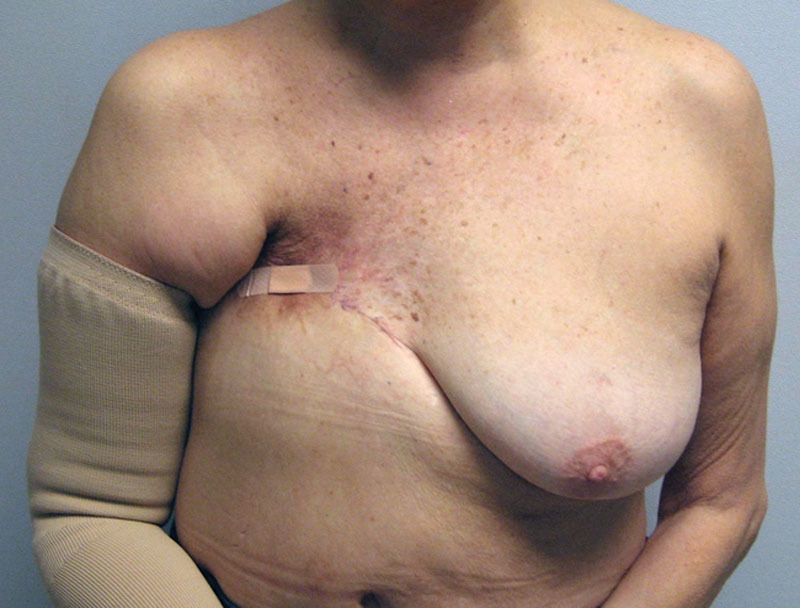
Postoperative photograph following chest wall debridement and reconstruction with an LD musculocutaneous flap.

The LD musculocutaneous flap only cohort had a 14.3% surgical revision rate, compared with 42.9% in the 1-stage with implant, 50% in the 2-stage with expander/implant, and 33.3% in the 3-stage with expander/implant cohorts (Table 3; *p* = 0.135). The rate of contralateral symmetry procedures was 10.7%, 0%, 25%, and 6.7%, across the 4 cohorts, respectively (*P* = 0.410). Figures 2–4 illustrate a patient who had a bilateral 3-stage LD musculocutaneous flap breast reconstruction. Figures 5 and 6 illustrate a patient who had an LD musculocutaneous flap without a prosthetic device.

**Table 3.** Revision Rates Following 1-, 2-, and 3-Stage Latissimus Dorsi Musculocutaneous Flap Reconstruction

	1-Stage Latissimus Only n = 28 48.30%	1-Stage Latissimus with Implant n = 7 12.10%	2-Stage Latissimus with TE/Implant n = 8 13.80%	3-Stage Latissimus with Delayed TE/Implant n = 15 25.90%	*P*
Surgical revision	4 (14.3%)	3 (42.9%)	4 (50%)	5 (33.3%)	0.135
Contralateral symmetry procedure	3 (10.7%)	0 (0%)	2 (25%)	1 (6.7%)	0.41

## DISCUSSION

Breast reconstruction has evolved the last few years, and the number and variety of options has increased. Prosthetic reconstruction remains the most common option for women following mastectomy; however with the advent of microsurgery, autologous free flap-based options are now offered with increased popularity and include a variety of donor sites.^15–18^ Prosthetic reconstruction is currently performed as a 2-stage process^19^ or as a 1-stage process^20^ Device placement varies between the partial and total pectoral muscle coverage as well as the prepectoral techniques.^21^ An alternative to total breast reconstruction is breast conservation that may or may not include oncoplastic reconstruction.^22,23^

All the abovementioned options are attractive approaches to breast reconstruction; however, reconstruction becomes challenging in the setting of primary reconstructive failure or tumor recurrence.^9,11,12^ This is exacerbated in the presence of prior RT or infection. The irradiated chest is subject to damage of the local tissue, and this effect can persist for many years.^24^ Subsequent reconstructive attempts following RT are also fraught with complications.^25^ It is this cohort of patients who have failed prior reconstruction due to infection, flap failure, and prior RT that are considered ideal candidates for the 1-, 2-, or 3-stage approach with the LD musculocutaneous flap.

The LD musculocutaneous flap has advantages and disadvantages. The technical aspects of raising, elevating, and harvesting the LD flap are straightforward and have been previously described.^26^ The vascular pedicle is reliable, a microsurgical anastomosis is not usually required, and there is minimal long-term functional loss associated with use of the LD muscle.^27^ The primary limitation of the LD musculocutaneous flap is that the amount of tissue is sometimes limited, and a prosthetic device is sometimes considered for volume augmentation.^27^ An alternative method used to augment the volume of the LD musculocutaneous flap while avoiding an implant is immediate fat grafting.^28,29^ Fat is injected into the pectoralis muscle, as well as the latissimus and the subcutaneous tissue of the skin paddle. Demiri et al^29^ reported the outcomes of 23 patients undergoing the mean volume of 406 cm^3^ of fat graft, with 70% of patients requiring more than one sessions of fat grafting and compared this group to 24 patients undergoing LD and implant reconstruction. The implant group had a higher postoperative morbidity profile with 8% of implant extrusion and 54% of Baker II/IV capsular contracture, whereas the fat grafting group had only one episode of dehiscence. Furthermore, when considering limitations of the LD flap, donor site seroma is relatively common and there may be some restriction of range of motion for the first postoperative year.^24,27,30^

Given that we now perform the majority of breast reconstruction in this era of muscle preservation (perforator flaps and prepectoral placement of devices), the frequency of the LD musculocutaneous flap for primary reconstruction has declined; however, its use for secondary reconstruction has remained a viable option. Its benefits serving this role have been studied extensively and demonstrated to decrease the rates of complication compared with purely prosthetic-based reconstruction.^10,30,31^ The introduction of well-perfused, pliable soft tissues may mitigate the adverse effects of radiation, improving wound healing and decreasing risks of capsular contracture.^10,30^ Overall, patients benefit from salvage rates as high as nearly 95%^31^ in addition to the potential for very good to excellent aesthetic results.^10^

In this study, the role of the LD musculocutaneous flap in salvage breast reconstruction was thoroughly evaluated. The following 4 distinct cohorts using the LD flaps were studied: (1) LD flap alone, (2) LD flap and simultaneous prosthetic device, (3) LD flap and delayed implant, and (4) LD flap and delayed TE/implant reconstruction. The present algorithm (Fig. 1) bases decision making on both the severity of the radiation injury and the specific reconstructive needs of the patient to maximize aesthetic outcomes and minimize the risk of complications and reconstructive failure.

Overall, the data support the efficacy for this approach, with fairly low rates of postoperative complications across all cohorts (Table 2). Even in those patients with considerable radiation injury, the 3-stage approach resulted in only 1 infection out of 15 patients and no reoperations or reconstructive failures. Only 2 patients in the 1- and 2-stage latissimus with implant cohorts experienced reconstructive failure and explantation. The 2-stage patient developed and infected seroma of her TE that was removed after the completion of expansion and exchanged for a permanent implant after resolution of the infection. The 1-stage patient had her implant removed and elected for no additional reconstruction.

From an aesthetic perspective, the use of the LD musculocutaneous flap allows for the recruitment of additional skin to mitigate the tightening and fibrotic effects of the chest wall radiation and maximize breast symmetry. Placement of a TE immediately or on a delayed basis can further expand the skin envelope to provide additional surface area. The expanded skin envelope allows for a larger implant and more natural, ptotic shape that closer resembles the native breast and in some cases may obviate the need for a contralateral symmetry procedure (Table 3). Although fat grafting may provide further benefits in the setting of a radiated field,^32^ only a minority of patients underwent surgical revision or fat grafting (Table 3).

In conclusion, this study has demonstrated that in the setting of prior RT and primary reconstructive failure, it is important to provide an option that will be predictable and reliable and have a high rate of reconstructive success. Because most of these patients have had reconstructive failure, the goal was to provide an appropriate number of procedures to achieve success and minimize adverse events given the complexity associated with local soft tissues. The goal was not necessarily to duplicate the normal breast or to approach the quality of an immediate reconstruction but rather to create a breast mound that resembles a true breast in terms of form. Staging the operation is another way to further reduce complications.
